# Zero gravity induced by parabolic flight enhances automatic capture and weakens voluntary maintenance of visuospatial attention

**DOI:** 10.1038/s41526-021-00159-3

**Published:** 2021-07-27

**Authors:** Adriana Salatino, Claudio Iacono, Roberto Gammeri, Stefano T. Chiadò, Julien Lambert, Dominika Sulcova, André Mouraux, Mark S. George, Donna R. Roberts, Anna Berti, Raffaella Ricci

**Affiliations:** 1 Department of Psychology, University of Turin, Turin, Italy; 2 Institute of Neuroscience (IoN), Université Catholique de Louvain Brussels, Brussels, Belgium; 3 Vastalla S.r.l., Turin, Italy; 4 Department of Psychiatry and Behavioral Sciences, Medical University of South Carolina, Charleston, SC USA; 5 Ralph H. Johnson VA Medical Center, Charleston, SC USA; 6 Department of of Radiology and Radiological Science, Medical University of South Carolina, Charleston, SC USA

**Keywords:** Neuroscience, Neurological manifestations, Human behaviour

## Abstract

Orienting attention in the space around us is a fundamental prerequisite for willed actions. On Earth, at 1 g, orienting attention requires the integration of vestibular signals and vision, although the specific vestibular contribution to voluntary and automatic components of visuospatial attention remains largely unknown. Here, we show that unweighting of the otolith organ in zero gravity during parabolic flight, selectively enhances stimulus-driven capture of automatic visuospatial attention, while weakening voluntary maintenance of covert attention. These findings, besides advancing our comprehension of the basic influence of the vestibular function on voluntary and automatic components of visuospatial attention, may have operational implications for the identification of effective countermeasures to be applied in forthcoming human deep space exploration and habitation, and on Earth, for patients’ rehabilitation.

## Introduction

Spatial attention on Earth, i.e. the capacity to allocate our attentional resources to specific regions in the space around us, enhances our ability to select relevant objects and to anticipate expected events, in order to coherently act in our environment. Neuropsychological studies in stroke patients with spatial awareness impairments and in patients with vestibular disorders, together with vestibular manipulations in healthy individuals, show that orienting attention in space not only requires the interaction between automatic (i.e. bottom-up, stimulus-driven, or exogenous) and voluntary (i.e. top-down, goal-directed, or endogenous) components of visuospatial attention^1–4^ but also engages the vestibular system^5–7^. However, the specific contribution of vestibular signals to automatic and voluntary attention remains largely unknown.

On Earth, the vestibular system, situated in the inner ear, encodes head position and enables postural balance by computing the crystals orientation in otolith organs with respect to gravity. In zero gravity, the weight of otolith (utricular) afferent inputs is absent or greatly reduced^8,9^. Thus zero gravity represents a unique condition to unweight the otolith organ and test its contribution to visuospatial attention. Studies in microgravity induced by free-fall conditions of parabolic and orbital space flights (see^9^), suggested that this condition increases the weight of visual information over vestibular signals, enhancing visual processing^10,11^. This in turn might affect attentional processes. However, the evidence on how microgravity might affect visuospatial attention is scant and controversial^10–12^ and no data exist on its effects on the two distinct attentional components (automatic vs. voluntary).

**Fig. 1:**
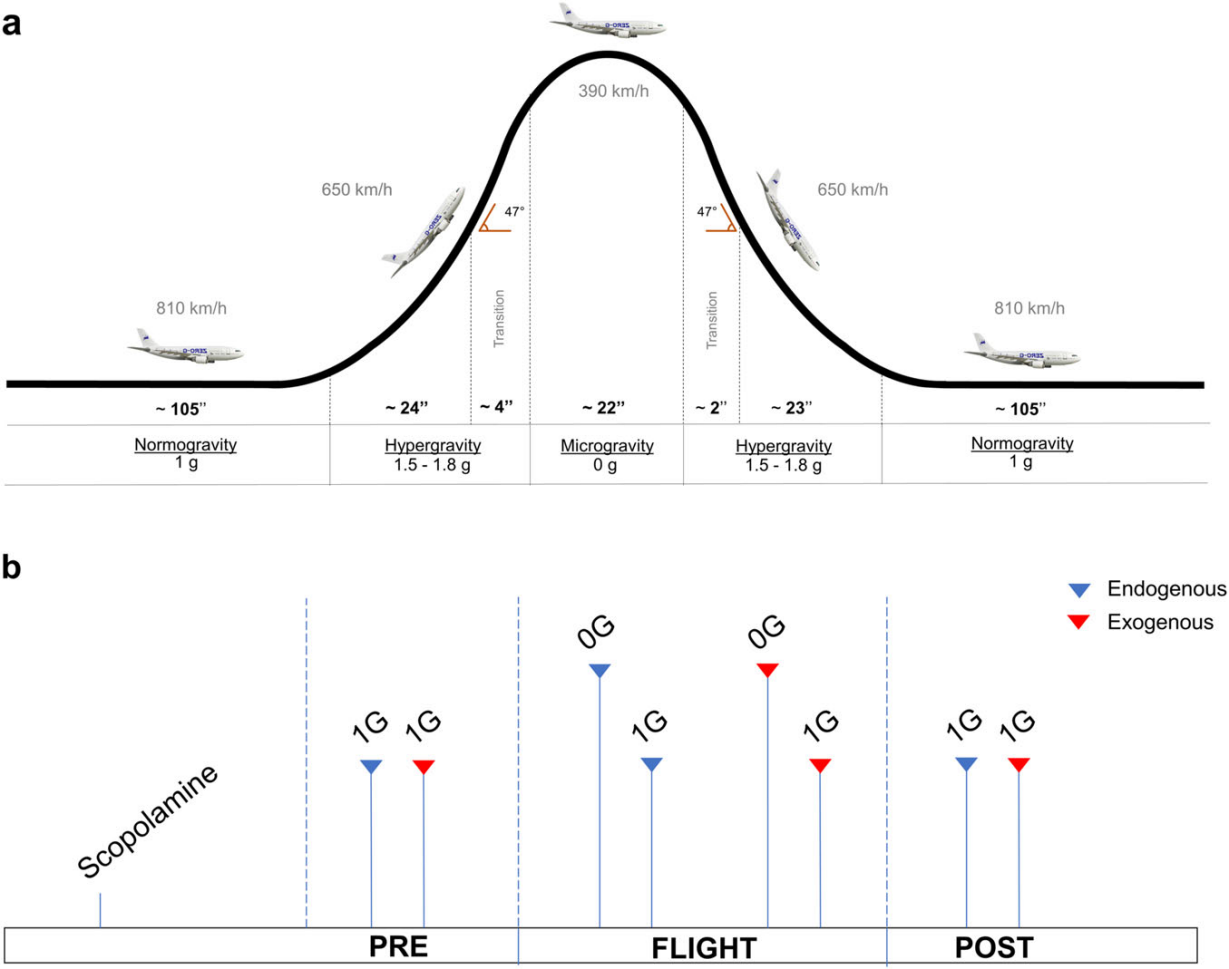
Overview of our parabolic flight experiment. Flight trajectory during the parabolic manoeuvre (**a**) and study timeline (**b**).

We took advantage of short periods of microgravity (~22 s), obtained during parabolic flights (72nd ESA Parabolic Flights Campaign, Fig. 1a), to investigate whether temporary unweighting of the otolith organ may differently affect automatic and voluntary orienting of visuospatial attention. Because in microgravity there is a decrease of vestibular weight in favour of vision, we predicted that increased saliency of visual stimuli might boost automatic capture of stimulus-driven attention, without affecting spatial orienting of voluntary attention, which is driven by internal goals.

To test these hypotheses we asked seven healthy participants to perform modified versions of the cue-to-target Posner task^4^ in which subjects had to detect visual targets, appearing in their left or right hemifield. In Posner’s procedures, central or peripheral visual cues indicate the position of the upcoming target. It is well established that peripheral, non-informative (i.e. non-predictive) cues orient attention exogenously, whereas central informative (i.e. predictive) cues orient attention endogenously^4^. In our study, peripheral cues exogenously captured attention, while central cues endogenously oriented attention. In valid trials, the cue indicated where the target would actually appear. In invalid trials, the cue indicated the side opposite to the upcoming target. Subjects’ reaction times (RTs) are typically faster in valid than in invalid trials (i.e. ‘validity effect’). Depending on the different types of trials, this procedure allowed measuring the engagement of attention to the target, disengagement of attention from its current focus, and attentional shift. Participants’ performed the tasks in the following *conditions*: 1 g before (PRE) flight, 0 g (0 G) and 1 g (1 G) during flight, 1 g after (POST) flight (Fig. 1b, Methods).

## Results

### Accuracy

One participant suffered from motion sickness during the first block of parabolas and his data were not considered in the analysis (see details in the Procedure). Participants were highly accurate in both the *exogenous* (mean accuracy > 94%) and the *endogenous* task (mean accuracy > 93%), as indexed by the high percentage of correct responses (see Supplementary Table 1 for details). The repeated-measures ANOVAs on participants’ accuracy for both tasks did not show any significant effect or interaction.

### Reaction times

**Fig. 2:**
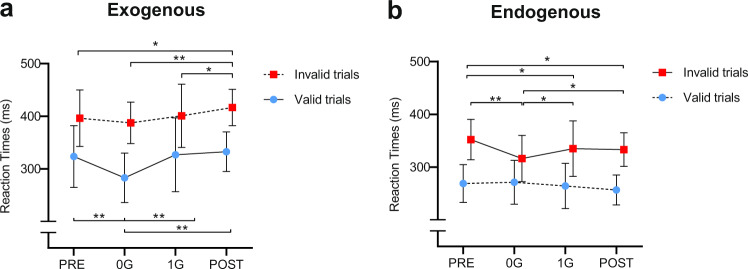
Mean reaction times (RTs) and SD (*n* = 5) for valid and invalid trials for the two tasks across gravity conditions. **a** *Exogenous* task: valid trials were faster in 0 g compared to 1 g conditions; invalid trials were slower in POST compared to all other conditions. **b** *Endogenous* task: invalid trials were faster in 0 g compared to 1 g conditions; invalid trials were slower in PRE compared to 1 G and POST. There were no significant differences for valid trials. Newman-Keuls post-hoc analyses, *P < 0.05 and **P < 0.01. PRE = 1 g before flight, 0 G = 0 g on the flight, 1 G = 1 g on the flight, POST = 1 g after the flight.

By measuring participants’ RTs, we observed a *validity effect* (i.e. faster RTs for valid compared to invalid trials) for both *exogenous* [*F*_1,4_ = 90.149; *P* = 0.001; partial *η*^2^ = 0.958] and *endogenous* [*F*_1,4_ = 18.712; *P* = 0.012; partial *η*^2^ = 0.824] tasks and a significant *validity by condition* interaction for both tasks. Consistently with the previous studies^4^, participants showed faster RTs for valid trials compared to invalid trials for both *exogenous* (valid: mean = 328.58 ± 51.19; invalid: mean = 388.39 ± 41.35) and *endogenous* (valid: mean = 272.01 ± 35.36; invalid: mean = 331.32 ± 40.05) tasks. Interestingly, Newman–Keuls post hoc analyses^13^ revealed, for the interaction of the *exogenous* task [*F*_3,12_ = 4.688; *P* = 0.022; partial *η*^2^ = 0.540], faster RTs for valid trials in 0 G compared to PRE (*P* = 0.0002), 1 G (*P* = 0.0003), and POST (*P* = 0.0002). In addition, invalid trials were slower in POST compared to PRE (*P* = 0.027), 0 G (*P* = 0.005), and 1 G (*P* = 0.040). For the interaction of the *endogenous* task [*F*_3,12_ = 6.809; *P* = 0.006; partial *η*^2^ = 0.630], Newman–Keuls post hoc analyses showed faster RTs for invalid trials in 0 G compared to PRE (*P* = 0.0008), 1 G (*P* = 0.033), and POST (*P* = 0.022), besides, invalid trials were slower in PRE compared to 1 G (*P* = 0.020) and POST (*P* = 0.030). No significant differences were found for valid trials (Fig. 2).

**Fig. 3:**
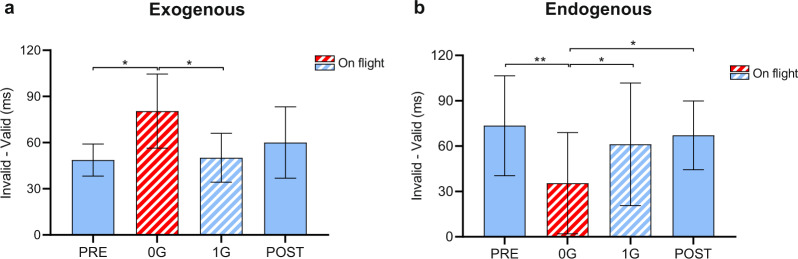
Validity effect for the two tasks across conditions. Mean values and SD (*n* = 5) are reported. **a** E*xogenous* task: the validity effect was larger in 0 g compared to PRE and 1 G, and there was a similar trend for POST (*P* = 0.055); **b** *endogenous task*: the validity effect was smaller in 0 g compared to all 1 g conditions. Newman–Keuls post hoc analyses, *P ≤ 0.05 and **P ≤ 0.01. PRE = 1 g before the flight, 0 G = 0 g on the flight, 1 G = 1 g on the flight, POST = 1 g after the flight.

When looking at the validity effect (invalid—valid trials), Newman–Keuls post hoc analyses showed, for the *exogenous* task, a larger *validity effect* in 0 G compared to PRE (*P* = 0.027) and 1 G (*P* = 0.021) conditions, and a trend in the same direction compared to POST (*P* = 0.055). This last finding may be explained by the increased RTs of invalid trials in the POST condition with respect to all other conditions, likely due to fatigue effects. In contrast, for the interaction of the *endogenous* task, post hoc analyses revealed a smaller *validity effect* in 0 G compared to PRE (*P* = 0.006), 1 G (*P* = 0.015), and POST (*P* = 0.011) conditions (Fig. 3 and Supplementary Fig. 1).

To sum up, our results show that microgravity increased the effect of exogenous cueing, but decreased the effect of endogenous cueing, speeding up the engagement of automatic attention by peripheral cues in the exogenous task and disengagement of voluntary attention in the endogenous task.

## Discussion

The observation of the validity effect in microgravity indicates that attentional systems still operate when unweighting the otolith organs. The finding that microgravity increased the validity effect for the exogenous cueing, but decreased it for the endogenous cueing, strongly suggests that otolith signals differentially affect the two attentional systems. The analyses revealed that 0 g speeded up attentional *engagement* to *valid* exogenously cued locations (targets), while quickening *disengagement* from *invalid* endogenously cued locations. In other words, 0 g might have enhanced, as we predicted, stimulus saliency and, consequently, stimulus-driven attentional capture, in the exogenous task. Conversely, the results of the endogenous task suggest that 0 g might have impaired the maintenance of voluntary attention at cued locations. These data indicate that microgravity, rather than enhancing the overall visual performance, might boost attention to peripheral salient visual stimuli with a resulting detriment of top-down control. It is worth noting that in the present study, attentional processes in 1 g were studied before and after 0 g, ruling out the impact of various dynamic processes, such as, for example, adaptation to parabolic flight (PF), practice with the task, or scopolamine pharmacodynamics.

The finding that microgravity differentially affects automatic and voluntary attention is consistent with the evidence of separate circuits underlying voluntary and automatic components of spatial attention^3,14^ and the existence of distinct vestibular projections reaching different structures of the two attention systems. A bilateral dorsal network (including the intraparietal sulcus and frontal eye fields) is mainly involved in goal-directed attention, while a right-lateralized ventral network (including the temporoparietal junction or TPJ, and the ventral frontal cortex) in stimulus-driven attention^14–17^. Some areas of the vestibular system overlap with posterior regions of the *ventral network*, such as TPJ and areas, such as supramarginal gyrus and angular gyrus^5,18,19^, which mainly process the space/environment around us using *egocentric* reference frames, i.e. centred on the viewer’s head/trunk/eyes^20^. Other vestibular projections reach the intermediate layers of the superior colliculus (iSC), which carry out multisensory integration in egocentric space and are engaged by the *dorsal attention system*^6,21^.

It is well-known that vestibular signals anchor visuospatial processing to egocentric frames of reference (see for example^22^). We may speculate that, in the present study, reduced otolith input to brain regions overlapping with the ventral attention system might have weakened egocentric reference frames^23^ in favour of allocentric reference frames (in which stimuli in space are represented in terms of coordinate systems independent of the subjects’ viewpoint), which are processed by more posterior and ventral areas (i.e. posterior inferior temporal and lateral occipital areas, see^20^). The reduced weight of target spatial position with reference to the subjects’ body/head might have led to stimulus distance underestimation and/or stimulus size overestimation (which are observed in microgravity^24^) enhancing stimulus salience and stimulus-driven attentional capture, as shown by findings of the exogenous task. Moreover, reduced otolith input to iSC might have weakened multisensory integration in egocentric space, impairing maintenance of voluntary attention at cued locations within this space, as suggested by finding of the endogenous task. Weakening of egocentric and strengthening of stimulus-centred coordinates systems, in 0 g, might have enhanced automatic attentional orienting to peripheral salient stimuli, with detriment effects on voluntary goal-directed attention. Since in our study egocentric and, allocentric reference frames were aligned, future studies disentangling the two coordinates systems (see for example ref. ^25^) are necessary to test and validate our hypothesis.

One of the limitations of PF experiments might be the unspecific effects of the weightlessness experience that may potentially distract the subject from task performance. However, distraction is expected to negatively affect the overall performance^26^—increasing errors and RTs of both valid and invalid trials in both types of exogenous and endogenous tasks—rather than selectively facilitating responses to valid exogenously cued locations (targets) and to invalid endogenously cued locations, as it occurred in our PF experiments.

Although these findings warrant further in-depth investigation, they may have operational implications for defining new countermeasures to be applied in forthcoming human deep space exploration and habitation^8,27^, where humans will experience different *g*. Indeed, the efficiency of human performance in microgravity is crucial to the success of long-term space stations and interplanetary missions^8,11,28,29^. We may speculate that enhanced reflexive attentional capture by upcoming environmental visual stimuli and weakened ability to voluntarily maintaining attention at specific regions of the egocentric space might contribute to some altered perceptions experienced by the astronauts during spaceflights^6,27,28^. For example, in microgravity astronauts misperceive objects size (i.e. height and depth overestimation) and objects distance (i.e. distance underestimation) and may manifest the illusion that the wall or the floor is looming over them when they are actually approaching it^24,30^ as if the enhanced visual salience of objects in the exogenous system prevails over the subjects’ voluntary control of viewer’s centred events. Moreover, strengthened automatic attentional orienting in 0 g compared to 1 g, might potentially explain the increased cognitive fatigue reported by astronauts when operating in microgravity^27,28^. Indeed, heightened automatic reorienting towards upcoming stimuli might distract from engagement in ongoing (goal-directed) activities, increasing the cognitive load.

By shedding new light on the basic influence of the vestibular function on attentional processes, the present findings may offer a theoretical framework not only for space research but also, on Earth, for the design of innovative cognitive rehabilitation strategies in individuals with vestibular disorders^30,31^ or with disorders of spatial awareness due to a stroke (such as unilateral spatial neglect and related disorders^32–35^).

## Methods

### Participants

Seven healthy right-handed volunteers (5 males and 2 females; mean age 35.83 ± 11.2 years; mean education = 19 years ± 1.4) with no previous experience with Parabolic Flights participated in the study. They had a normal or corrected-to-normal vision. Handedness was estimated using the Edinburgh Handedness Inventory^36^ test, which ranges from −100 (completely left-handed) to +100 (completely right-handed). All participants had passed the Parabolic Flight Medical Certificate (DM-2016- en-ed3) equivalent of an Air Force Class III medical examination. All participants provided a signed informed consent form. The study was approved by the Ethics Committees of the University of Turin (Italy) and the French Ethics Committee of the University of Caen Normandie (France) in compliance with French legislation and the Declaration of Helsinki for human participants. One participant suffered from motion sickness during the first block of parabolas and his data were not considered in the analysis (see the Statistical Analysis section for details). Thus the presented data come from 6 participants (4 males and 2 females; mean age 35.00 ± 11.90 years; mean education = 19 years ± 1.5; Edinburgh Handedness Inventory mean score = 76 ± 16.9).

### Procedure

**Fig. 4:**
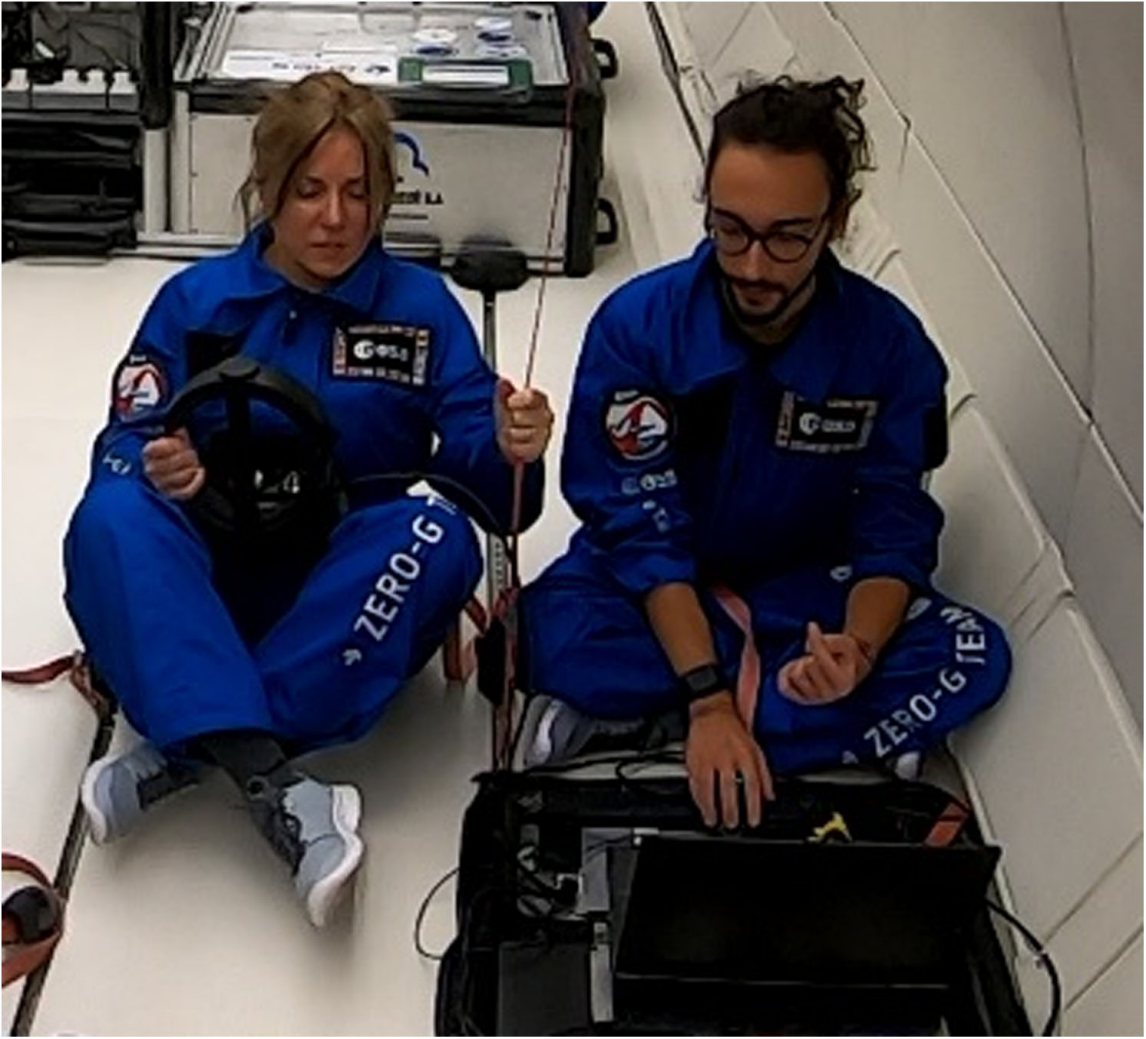
Photo of the experimental set-up in flight. The participant was sitting cross-legged and firmly strapped to the floor at 60 cm from the screen of a laptop that was attached on the floor and centred on the sagittal mid-plane. The experimenter in charge of administering the experimental task and supervising its execution was positioned on the right side of the participant, slightly further back so as not to interfere with task execution.

The experiment was performed during the 72nd European Space Agency (ESA) Parabolic Flights Campaign (PFC), in November 2019, onboard the Airbus A310 Zero-G and took place at Bordeaux-Merignac airport (France). The flights were run by Novespace (http://www.novespace.fr). One campaign consists of three parabolic flights (PFs) on three consecutive days. Each flight has a duration of two to three hours and includes 30 parabolic manoeuvres, divided into 6 series of 5 parabolas each. There is a 5 min (1 g) interval between the series of parabolas, with a longer interval (8 min) between the first three series and the last three series of parabolas. The reduced-gravity environment is obtained by carrying out, with the A310 Zero-G airplane, a PF manoeuvre (see Fig. 1a) producing periods of weightlessness for approximately 22 s. The air pressure in the cabin is maintained at approximately 800 millibars during the parabolas, which corresponds to an altitude of about 2000 m. The temperature is between 20 and 25 °C. Each parabola starts with a pull-up and ends with a pull-out at 1.8 g (hypergravity). These phases last about 23–24 s. On each flight, we tested two participants, each participant taking part in only one flight. They performed each attentional task (endogenous or exogenous) during the 0 g phase of a sequence of 5 parabolas and during the 1 g 5 min-interval following the same sequence of parabolas. Participants performed the two tasks during two consecutive series of parabolas with the order of the two tasks balanced across subjects. Since on each flight we had equipped only one experimental setting for the present study (a second experimental setting was equipped for a different study, here not reported), the two participants underwent the present study following a sequential order: the first participant performed the two attentional tasks during the first two series of five parabolas and the following 1 g intervals, whereas the second participant performed the study during the fourth and fifth series of five parabolas and the subsequent intervals. This order was counterbalanced across participants. During the flight, participants were sitting cross-legged and firmly strapped to the floor at 60 cm from the screen (17” monitor) of a laptop, that was attached to the floor and centred on their sagittal mid-plane. An operator in charge of administering the experimental task and supervising its execution was positioned on the right side of the subject, slightly further back so as not to interfere with task execution (Fig. 4). The authors affirm that human research participants provided informed consent, for publication of the image in Fig. 4.

Before the flight, participants received a muscarinic receptor antagonist (scopolamine: 0.25 mg/1 mL; 0.7 mL for males and 0.5 mL for females), known to alleviate motion sickness^37^. Nonetheless, one participant suffered from motion sickness during the first series of parabolas, during the performance of the endogenous task, and his data were not considered in the analysis. In the second series of parabolas, a backup participant replaced him, performing the exogenous task (following the scheduled order of task administration).

The exogenous and endogenous tasks were administered in four *conditions*, starting soon after the scopolamine injection (Fig. 1b): at 1 g before the parabolic flight (PRE), at 0 g (0 G) and 1 g (1 G) onboard the flight and at 1 g, soon after the flight (POST). During the ground sessions, the same setting of 0 g and 1 g in-flight sessions were reproduced, i.e. participants were sitting cross-legged on the floor at 60 cm from the screen of an identical laptop, with the operator sitting in the same position as during the flight. Visual stimuli presentation and data collection were performed using E-prime software. The order of exogenous and endogenous conditions was counterbalanced across subjects.

### Exogenous attention task

**Fig. 5:**
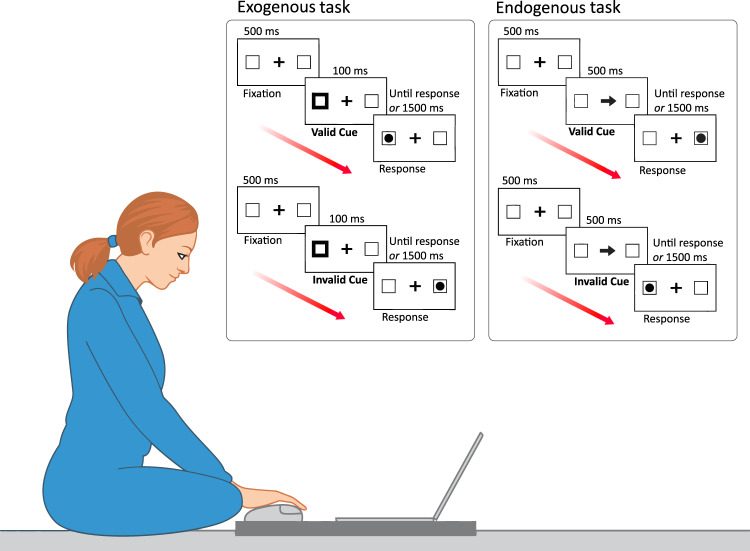
Schematic of the experimental set-up. Examples of visual stimuli (original proportions are not preserved in the figure), and the experimental timeline for the Exogenous and Endogenous Tasks.

The sequence of experimental events is presented in Fig. 5. Each trial began with a central fixation cross (size: 0.4° × 0.4°) and two lateral boxes (size: 1° × 1°), centred 8° of visual angle to the left and the right of the central fixation. This “Fixation” period lasted 500 ms and was followed by a “Cue” period. Cues consisted of a thickening (from 0.05° to 0.14°) of the contour of one box. Stimulus onset asynchrony (SOA) between cue and target was 100 ms, with the visual target preceded by a valid cue (signalling where the target could appear) or an invalid cue (signalling the spatial position opposite to where the target could appear). Participants were asked to report, as quickly and accurately as possible, the presence of a peripheral visual target (a dot, 0.3° in diameter), presented inside of one of the two boxes, by pressing the left or right button of the mouse. The mouse was fixed on the floor in front of the participant, centred on her/his sagittal midplane. The target remained visible until a response was made or until 1500 ms had elapsed. Participants were instructed to respond only to the targets and not to the orienting cues. They were asked to keep their eyes on the central fixation cross during the entire block of trials^38^. It is well established that peripheral, non-informative (non-predictive) cues orient attention exogenously^4^. For this reason, valid trials were 50% of all trials, as it is done in this type of paradigm^2,4^. Participants were informed about the non-informative value of the cues.

Stimuli were administered in sessions of five blocks. Within each block 8 valid and 8 invalid trials were presented in random order. For the 0 G condition, each block was administered in correspondence of the parabola 0 g phase and for the 1 G condition, the 5 blocks were presented during the 5 min interval occurring between series of parabolas. Participants’ RTs and accuracy were recorded and constituted the dependent variables.

### Endogenous attention task

The sequence of experimental events is presented in Fig. 5. Each trial began with a central fixation cross (size: 0.4° × 0.4°) and two lateral boxes (size: 1° × 1°), presented at 8° of visual angle to the left and the right of the central fixation cross. The “Fixation” period lasted 500 ms and was followed by a “Cue” period in which an arrow took the place of the central fixation cross. The SOA between cue and target was 500 ms, with the visual target preceded by a valid (signalling where the target could appear) or an invalid (signalling the spatial position opposite to where the target could appear) cue. Participants were asked to report, as quickly as possible and accurately as possible, the presence of a peripheral visual target (a dot, 0.3° in diameter) presented inside one of the two boxes, by pressing the left or right button of the mouse that was fixed on the floor. The target remained visible until a response was made or until 1500 ms had elapsed. Participants were instructed to respond only to the targets and not to the orienting cues. They were also asked to keep their eyes on the central fixation cross during the entire block of trials^38^. It is well established that central informative (i.e. predictive) cues orient attention endogenously^4^. To make the cue informative, trials were 80% valid and 20% invalid, as it is done in this type of experimental paradigm^2,4^. Participants were informed about the informative value of the cues. Stimuli were presented in sessions of 5 blocks. Within each block, 14 trials were presented in random order. For the 0 G condition, each block was administered in correspondence of the 0 g phase and for the 1 G condition, the 5 blocks were presented during the 5-min interval occurring between series of parabolas. Participants’ Reaction Times and Accuracy were recorded and constituted the dependent variables.

### Statistical analysis

For each participant, RTs faster than 100 ms (i.e. anticipatory responses) and slower than 1000 ms were classified as outliers^39^. For technical reasons, the data of one subject (S6) in the PRE condition of the *exogenous* task were not complete and therefore they were not included in the analyses. Given that also data of the *endogenous* task for the subject who got sick (S1) were not available (and the back-up subject S1_BK only performed the exogenous task), the main statistical analyses were performed on five subjects for all four gravity conditions of the *exogenous* and *endogenous* task.

For both the *exogenous* and *endogenous* tasks, data were normally distributed as assessed by the Shapiro–Wilk test. Therefore, we performed separate repeated-measures ANOVAs for the two tasks on accuracy and RTs for correct responses with *validity* (valid and invalid) and *condition* (PRE, 0 G, 1 G, POST) as within-subject factors. Statistical analyses were performed using SPSS (IBM, Version 26.0) and STATISTICA (Version 12.0) with alpha set at 0.05 (two-tailed). All data are presented as means with the SD. Effect sizes are indicated for significant effects.

### Reporting summary

Further information on research design is available in the Nature Research Reporting Summary linked to this article.

## Supplementary information

Supplementary Information

Reporting Summary

## Data Availability

The data that support the findings of this study are available from the corresponding authors upon reasonable request. The data are not publicly available due to containing information that could compromise research participant privacy. Please email authors to request de-identified data and we will respond to any reasonable requests.
