# Supplementary material for: Zero gravity induced by parabolic flight enhances automatic capture and weakens voluntary maintenance of visuospatial attention
Source: NPJ Microgravity. 2021 Jul 27;7:29. doi: 10.1038/s41526-021-00159-3 (PMC8316350; doi:10.1038/s41526-021-00159-3)
Supplement: Supplementary file 1 — Supplementary Information [file 41526_2021_159_MOESM1_ESM.pdf]

Zero gravity induced by parabolic flight enhances automatic capture and weakens voluntary maintenance of visuospatial attention

Supplementary Results and Note

Adriana Salatino *[1,2], Claudio Iacono [1†], Roberto Gammeri [1†], Stefano T. Chiadò [3], Julien Lambert [2], Dominika Sulcova [2], André Mouraux [2], Mark S. George [4,5], Donna R. Roberts [6], Anna Berti [1], Raffaella Ricci *[1,4]

1. Department of Psychology, University of Turin, Turin, Italy

2. Institute of Neuroscience (IoN), Université Catholique de Louvain Brussels, Belgium

3. Vastalla S.r.l., Turin, Italy

4. Department of Psychiatry and Behavioral Sciences, Medical University of South Carolina, Charleston, SC, USA

5. Ralph H. Johnson VA Medical Center, Charleston, SC USA

6. Department of of Radiology and Radiological Science, Medical University of South Carolina, Charleston, SC, USA

†Contributed equally to this work

**Corresponding Author: Raffaella Ricci, Department of Psychology, University of Turin, Turin, Italy, Via Verdi 10, 10124 Turin, Italy. Email: raffaella.ricci@unito.it*

Adriana Salatino, Institute of Neuroscience (IoN), Université Catholique de Louvain Brussels, Avenue Mounier, 54 1200 Brussels, Belgium. Email: adriana.salatino@uclouvain.be

Supplementary Table 1. Individual data for the Exogenous and the Endogenous tasks.

EXOGENOUS

		**PRE**		**0G**		**1G**		**POST**	
		VALID	INVALID	VALID	INVALID	VALID	INVALID	VALID	INVALID
**S1_ BK**	RT (ms)	*417.7 ± 92.7*	*449.8 ± 105.2*	*337.1 ± 123.7*	*383.0 ± 60.2*	*425.6 ± 131.1*	*465.1 ± 136.1*	*384.2 ± 61.7*	*439.0 ± 42.8*
	Acc (%)	*100*	*100*	*100*	*90*	*100*	*97.5*	*100*	*97.5*
	Outliers	*1*	*0*	*0*	*0*	*0*	*0*	*0*	*0*
**S2**	RT (ms)	*313.4 ± 59.6*	*358.7 ± 44.7*	*310.0 ± 84.5*	*377.7 ± 76.9*	*295.6 ± 46.8*	*336.9 ± 46.6*	*311.4 ± 56.2*	*361.5 ± 42.2*
	Acc (%)	*100*	*87.5*	*100*	*100*	*100*	*100*	*100*	*100*
	Outliers	*0*	*0*	*0*	*1*	*0*	*0*	*0*	*0*
**S3**	RT (ms)	*364.3 ± 65.4*	*423.3 ± 65.3*	*334.5 ± 152.1*	*427.5 ± 145.0*	*398.2 ± 69.0*	*441 ± 72.7*	*373.6 ± 87.1*	*402.7 ± 67.8*
	Acc (%)	*95*	*100*	*100*	*92.5*	*95*	*97.5*	*95*	*97.5*
	Outliers	*0*	*0*	*0*	*0*	*0*	*0*	*0*	*0*
**S4**	RT (ms)	*321.4 ± 61.9*	*375.7 ± 83.3*	*264.5 ± 75.0*	*372.4 ± 83.4*	*315.1 ± 101.1*	*363.5 ± 79.7*	*354.8 ± 144.3*	*438.9 ± 146.1*
	Acc (%)	*100*	*92.5*	*100*	*82.5*	*97.5*	*95*	*97.5*	*95*
	Outliers	*0*	*0*	*0*	*0*	*0*	*0*	*0*	*0*
**S5**	RT (ms)	*260.2 ± 73.7*	*313.4 ± 43.5*	*229.5 ± 66.5*	*317.1 ± 60.2*	*260.1 ± 103.8*	*338.1 ± 57.6*	*298.8 ± 85.6*	*380.9 ± 67.2*
	Acc (%)	*92.5*	*65*	*97.5*	*67.5*	*97.5*	*75*	*97.5*	*74*
	Outliers	*4*	*3*	*1*	*1*	*0*	*2*	*1*	*0*
	Mean RT (ms)	*335.5 ± 58.8*	*384.2 ± 53.7*	*295.1 ± 46.8*	*375.5 ± 39.2*	*338.9 ± 70.1*	*389.0 ± 60.2*	*344.6 ± 37.7*	*404.6 ± 34.6*
	Acc (%)	*97.5*	*89*	*99.5*	*86.5*	*98*	*93*	*99*	*92.5*
	Sum Outliers	*5*	*3*	*1*	*2*	*0*	*2*	*1*	*0*

ENDOGENOUS

		PRE		0G		1G		POST	
		VALID	INVALID	VALID	INVALID	VALID	INVALID	VALID	INVALID
**S2**	RT (ms)	*263.5 ± 52.2*	*296.4 ± 47.1*	*265.1 ± 49.7*	*258.1 ± 55.5*	*250.5 ± 49.7*	*248.2 ± 35.9*	*237.4 ± 50.4*	*277.6 ± 41.2*
	Acc (%)	*96*	*100*	*96*	*100*	*96*	*95*	*96*	*95*
	Outliers	*1*	*0*	*0*	*0*	*0*	*0*	*0*	*1*
**S3**	RT (ms)	*259.5 ± 51.3*	*350.2 ± 94.0*	*293.4 ± 124.4*	*309.8 ± 84.6*	*261.3 ± 69.1*	*351.6 ± 79.3*	*264.4 ± 54.8*	*330.2 ± 47.9*
	Acc (%)	*100*	*90*	*100*	*100*	*96*	*85*	*96*	*90*
	Outliers	*0*	*0*	*0*	*0*	*0*	*0*	*0*	*0*
**S4**	RT (ms)	*272.8 ± 79.2*	*334.3 ± 72.4*	*270.8 ± 87.4*	*312.1 ± 110.1*	*301.1 ± 123.5*	*356.8 ± 106.9*	*283.4 ± 87.1*	*344.7 ± 122.6*
	Acc (%)	*100*	*95*	*98*	*75*	*98*	*90*	*100*	*80*
	Outliers	*1*	*0*	*1*	*2*	*3*	*3*	*0*	*0*
**S5**	RT (ms)	*245.2 ± 132.6*	*365.1 ± 216.9*	*223.8 ± 101.7*	*306.2 ± 162.1*	*216.0 ± 56.9*	*318.1 ± 204.1*	*233.0 ± 122.4*	*336.2 ± 93.2*
	Acc (%)	*100*	*85*	*94*	*85*	*94*	*75*	*100*	*60*
	Outliers	*1*	*2*	*1*	*1*	*9*	*2*	*9*	*4*
**S6**	RT (ms)	*337.8 ± 96.1*	*400.3 ± 106.0*	*337.2 ± 96.1*	*381.3 ± 93.5*	*325.3 ± 88.6*	*385.4 ± 139.4*	*298.3 ± 81.8*	*362.9 ± 135.2*
	Acc (%)	*100*	*100*	*100*	*100*	*100*	*100*	*100*	*95*
	Outliers	*0*	*0*	*0*	*1*	*0*	*0*	*1*	*1*
	Mean RT (ms)	*275.7 ± 36.0*	*349.3 ± 38.3*	*278 ± 41.5*	*313.5 ± 43.9*	*270.8 ± 42.9*	*332.0 ± 52.5*	*263.2 ± 28.3*	*330.3 ± 31.9*
	Acc (%)	*99.2*	*94*	*97.6*	*92*	*96.8*	*89*	*98.4*	*84*
	Sum Outliers	*3*	*2*	*2*	*4*	*12*	*5*	*10*	*6*

Legend: Reaction Times (RTs ± SD), Accuracy (Acc in %), and number of Outliers are reported for each experimental condition.

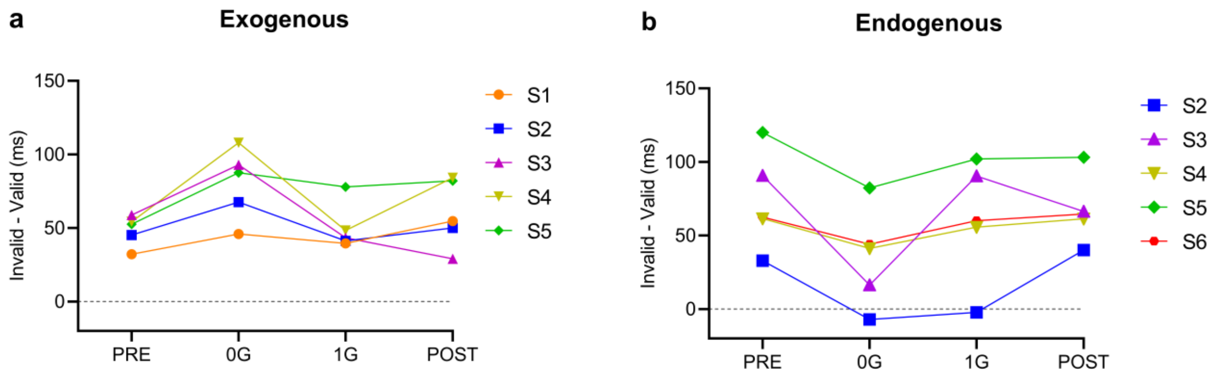

Supplementary Figure 1. Individual data of the validity effect (RTs for Invalid-Valid) for the Exogenous (a) and Endogenous (b) tasks across conditions.

Supplementary Note

As recently shown by neuroimaging studies, the human brain undergoes functional and structural neuroplastic changes after long-term periods of simulated (1-4) and real (5-10) microgravity. Neuroplastic brain changes are thought to occur as adaptive response to altered sensorimotor functioning (e.g., vestibular and proprioception senses and motor control), but also to brain unloading and fluids shift. While structural brain changes need time to occur, functional brain changes have also been reported during PF periods of weightlessness (11, 12) and following PF (13). PF functional changes, as measured by ERP signals (11) during PF or fMRI after PF (13), seem to mainly involve areas belonging to attentional networks, i.e. the right Supramarginal Gyrus, a structure belonging to TPJ (13). We may speculate that our findings might represent one of the behavioural correlates of PF functional changes. Future studies, concurrently collecting behavioural and neuroimaging measures, are necessary to unequivocally establish the neural bases of the observed cognitive effects.

Supplementary References

1. Roberts, D.R. et al. Cerebral cortex plasticity after 90 days of bed rest: data from TMS and fMRI. *Aviat Space Environ Med*, 81 1, 30-40 (2010) https://doi.org/10.3357/asem.2532.2009

2. Roberts, D.R., et al. (2015). Structural Brain Changes following Long-Term 6° Head-Down Tilt Bed Rest as an Analog for Spaceflight. *AJNR Am J Neuroradiol*, **36**, 2048 – 2054 https://doi.org/10.3174/ajnr.a4406

3. Cassady, K., Koppelmans, V., Reuter-Lorenz, P., Dios, Y.D., Gadd, N., Wood, S., Castenada, R.R., Kofman, I., Bloomberg, J., Mulavara, A., & Seidler, R. Effects of a spaceflight analog environment on brain connectivity and behavior. *NeuroImage*, **141**, 18-30 (2016) https://doi.org/10.1016/j.neuroimage.2016.07.029

4. Koppelmans, V. et al.. Brain plasticity and sensorimotor deterioration as a function of 70 days head down tilt bed rest. *PLoS One*, **12** (2017) https://doi.org/10.1371/journal.pone.0182236

5. Demertzi, A. et al. Cortical reorganization in an astronaut's brain after long-duration spaceflight. *Brain Struct Funct*, **221**, 2873 – 2876 (2015) https://doi.org/10.1007/s00429-015-1054-3

6. Roberts, D.R. et al. Effects of Spaceflight on Astronaut Brain Structure as Indicated on MRI. *N Engl J Med*, **377**, 1746–1753 (2017) https://doi.org/10.1056/NEJMoa1705129

7. Roberts, D.R., & Antonucci, M.. Effects of Spaceflight on Astronaut Brain Structure. *N Engl J Med*, **378** 6, 582-583 (2018) https://doi.org/10.1056/NEJMc1716067

8. Van Ombergen, A. et al.. Brain Tissue-Volume Changes in Cosmonauts. *N Engl J Med*, **379** 17, 1678-1680 (2018) https://doi.org/10.1056/NEJMc1809011

9. Van Ombergen, A. et al. Brain ventricular volume changes induced by long-duration spaceflight. *Proc Natl Acad Sci U S A*, **116**, 10531 – 10536 (2019) https://doi.org/10.1073/pnas.1820354116

10. Lee, J.K. et al.. Spaceflight-Associated Brain White Matter Microstructural Changes and Intracranial Fluid Redistribution. *JAMA Neurol*, **76**, 412–419 (2019) https://doi.org/10.1001/jamaneurol.2018.4882

11. Wollseiffen, P., Klein, T., Vogt, T., Abeln, V., & Schneider, S. Neurocognitive performance is enhanced during short periods of microgravity? Part 2. *Physiol Behav*, **207**, 48-54 (2019) https://doi.org/10.1016/j.physbeh.2019.04.021

12. Badran, B.W., Caulfield, K.A., Cox, C. et al. Brain stimulation in zero gravity: transcranial magnetic stimulation (TMS) motor threshold decreases during zero gravity induced by parabolic flight. npj Microgravity 6, 26 (2020). https://doi.org/10.1038/s41526-020-00116-6

13. Van Ombergen A, Wuyts FL, Jeurissen B, et al. Intrinsic functional connectivity reduces after first-time exposure to short-term gravitational alterations induced by parabolic flight. Sci Rep; 7(1):3061 (2017). https://doi.org/doi:10.1038/s41598-017-03170-5
