# Supplementary material for: Zero gravity induced by parabolic flight enhances automatic capture and weakens voluntary maintenance of visuospatial attention
Source: NPJ Microgravity. 2021 Jul 27;7:29. doi: 10.1038/s41526-021-00159-3 (PMC8316350; doi:10.1038/s41526-021-00159-3)
Supplement: Supplementary file 2 — Reporting Summary [file 41526_2021_159_MOESM2_ESM.pdf]

nature research

npj Microgravity NPJMGRAV-00606-T
Corresponding author(s): Prof Raffaella Ricci
Dr Adriana Salatino

Last updated by author(s): Jun 23, 2021

Reporting Summary

Nature Research wishes to improve the reproducibility of the work that we publish. This form provides structure for consistency and transparency in reporting. For further information on Nature Research policies, see our Editorial Policies and the Editorial Policy Checklist.

Statistics

For all statistical analyses, confirm that the following items are present in the figure legend, table legend, main text, or Methods section.

n/a	Confirmed	
☐	☒	The exact sample size (*n*) for each experimental group/condition, given as a discrete number and unit of measurement
☐	☒	A statement on whether measurements were taken from distinct samples or whether the same sample was measured repeatedly
☐	☒	The statistical test(s) used AND whether they are one- or two-sided<br>*Only common tests should be described solely by name; describe more complex techniques in the Methods section.*
☒	☐	A description of all covariates tested
☐	☒	A description of any assumptions or corrections, such as tests of normality and adjustment for multiple comparisons
☐	☒	A full description of the statistical parameters including central tendency (e.g. means) or other basic estimates (e.g. regression coefficient) AND variation (e.g. standard deviation) or associated estimates of uncertainty (e.g. confidence intervals)
☐	☒	For null hypothesis testing, the test statistic (e.g. *F*, *t*, *r*) with confidence intervals, effect sizes, degrees of freedom and *P* value noted<br>*Give P values as exact values whenever suitable.*
☒	☐	For Bayesian analysis, information on the choice of priors and Markov chain Monte Carlo settings
☐	☒	For hierarchical and complex designs, identification of the appropriate level for tests and full reporting of outcomes
☐	☒	Estimates of effect sizes (e.g. Cohen's *d*, Pearson's *r*), indicating how they were calculated

Our web collection on statistics for biologists contains articles on many of the points above.

Software and code

Policy information about availability of computer code

Data collection	Data were collected using E-prime Software
Data analysis	Statistical analyses were performed using SPSS (IBM, Version 26.0) and STATISTICA (Version 12.0) with alpha set at .05 (two-tailed).

For manuscripts utilizing custom algorithms or software that are central to the research but not yet described in published literature, software must be made available to editors and reviewers. We strongly encourage code deposition in a community repository (e.g. GitHub). See the Nature Research guidelines for submitting code & software for further information.

Data

Policy information about availability of data

All manuscripts must include a data availability statement. This statement should provide the following information, where applicable:

- Accession codes, unique identifiers, or web links for publicly available datasets
- A list of figures that have associated raw data
- A description of any restrictions on data availability

The data that support the findings of this study are available from the corresponding authors upon reasonable request. The data are not publicly available due to containing information that could compromise research participant privacy. Please email authors to request de-identified data and we will respond to any reasonable requests.

Field-specific reporting

Please select the one below that is the best fit for your research. If you are not sure, read the appropriate sections before making your selection.

☒ Life sciences ☐ Behavioural & social sciences ☐ Ecological, evolutionary & environmental sciences

For a reference copy of the document with all sections, see nature.com/documents/nr-reporting-summary-flat.pdf

Life sciences study design

All studies must disclose on these points even when the disclosure is negative.

Sample size	7 subjects took part in the study. One participant has been excluded from the study since he did not complete the experiment because he suffered from motion sickness during the first block of parabolas.<br>No statistical methods were used to predetermine sample size, but our sample size is similar to those reported in previous publications on Parabolic Flight. We enrolled this sample size due to the limitation on the number of zero gravity fliers.
Data exclusions	The data of one participant were excluded from the analyses since he did not complete the experiment because he suffered from motion sickness during the first block of parabolas. Data of one subject in the first the ground condition could not be collected for technical reasons.
Replication	Overall, all effects of measured behavior were replicated in the four conditions for each participant.
Randomization	Not applicable as no experimental groups were assigned. Randomization of block and trials was used for all participants. Participants performed the two tasks (exogenous and endogenous) during two consecutive series of parabolas with the order of the two tasks balanced across subjects. Stimuli were administered in sessions of 5 blocks. Within each block 8 valid and 8 invalid trials were presented in a random order.
Blinding	Investigators were not blind to allocation during experiment, since experiments were within subject comparisons and no experimental groups were assigned.

Reporting for specific materials, systems and methods

We require information from authors about some types of materials, experimental systems and methods used in many studies. Here, indicate whether each material, system or method listed is relevant to your study. If you are not sure if a list item applies to your research, read the appropriate section before selecting a response.

Materials & experimental systems

n/a	Involved in the study	
☒	☐	Antibodies
☒	☐	Eukaryotic cell lines
☒	☐	Palaeontology and archaeology
☒	☐	Animals and other organisms
☐	☒	Human research participants
☒	☐	Clinical data
☒	☐	Dual use research of concern

Methods

n/a	Involved in the study	
☒	☐	ChIP-seq
☒	☐	Flow cytometry
☒	☐	MRI-based neuroimaging

Human research participants

Policy information about studies involving human research participants

Population characteristics	Seven healthy subjects participated in the study, 5 males and 2 female (mean ages: 35 years). All participants were right handed and had normal vision.
Recruitment	Recruited via word of mouth within the University of Turin community in Turin, Italy and the Université Catholique de Louvain community in Brussels, Belgium
Ethics oversight	The study was approved by the French Ethics Committee of the University of Caen Normandie (France) and the Ethics committees of the University of Turin (Italy) and in compliance with French legislation and the Declaration of Helsinki for human participants.

Note that full information on the approval of the study protocol must also be provided in the manuscript.
